# Effect of HSV-IL12 Loaded Tumor Cell-Based Vaccination in a Mouse Model of High-Grade Neuroblastoma

**DOI:** 10.1155/2016/2568125

**Published:** 2016-08-17

**Authors:** David F. Bauer, Larisa Pereboeva, G. Yancey Gillespie, Gretchen A. Cloud, Osama Elzaafarany, Catherine Langford, James M. Markert, Lawrence S. Lamb Jr.

**Affiliations:** ^1^Department of Surgery, The University of Alabama at Birmingham School of Medicine, Birmingham, AL 35294, USA; ^2^Department of Medicine, The University of Alabama at Birmingham School of Medicine, Birmingham, AL 35294, USA; ^3^Department of Biostatistics and Preventive Medicine, The University of Alabama at Birmingham School of Medicine, Birmingham, AL 35294, USA

## Abstract

We designed multimodal tumor vaccine that consists of irradiated tumor cells infected with the oncolytic IL-12-expressing HSV-1 virus, M002. This vaccine was tested against the syngeneic neuroblastoma mouse model Neuro 2a injected into the right caudate nucleus of the immunocompetent A/J mice. Mice were vaccinated via intramuscular injection of multimodal vaccine or uninfected irradiated tumor cells at seven and 14 days after tumor establishment. While there was no survival difference between groups vaccinated with cell-based vaccine applied following tumor injection, a premunition prime/boost vaccination strategy produced a significant survival advantage in both groups and sustained immune response to an intracranial rechallenge of the same tumor. The syngeneic but unrelated H6 hepatocellular tumor cell line grew unrestricted in vaccinated mice, indicative of vaccine-mediated specific immunity to Neuro 2a tumors. Longitudinal analyses of tumor-infiltrating lymphocytes revealed a primary adaptive T cell response involving both CD4+ and CD8+ T cell subsets. Spleen cell mononuclear preparations from vaccinated mice were significantly more cytotoxic to Neuro 2a tumor cells than spleen cells from control mice as demonstrated in a four-hour* in vitro* cytotoxicity assay. These results strongly suggest that an irradiated whole cell tumor vaccine incorporating IL-12-expressing M002 HSV can produce a durable, specific immunization in a murine model of intracranial tumor.

## 1. Introduction

Neuroblastoma (NB) is the most common extracranial tumor diagnosed in children. Widely accepted standard therapy for high-risk NB includes 5 to 7 cycles of intensive cytotoxic chemotherapy, surgery, consolidative autologous stem cell transplantation (SCT), radiation therapy, and maintenance immunotherapy with anti-GD2 antibodies [1]. Such therapy carries considerable toxicity while survival remains generally poor as NB accounts for 15% of deaths attributable to cancer in childhood. Immunotherapy, in particular the use of tumor cell-based vaccines, is an attractive way of generating antineuroblastoma immunity and does not increase the toxicity of concurrent radio- or chemotherapy [2, 3].

We have generated a series of cell-based vaccines by combining tumor cells with replication-competent HSV oncolytic virus, which have demonstrated a host immune response following intratumor injection. Based on these findings, we sought to determine the feasibility of an oncolytic HSV-based whole cell peripheral vaccine against an intracranial tumor. We designed a whole cell tumor vaccine to incorporate the oncolytic IL-12-expressing replication-competent HSV-1 M002 into the Neuro 2a (N2a) neuroblastoma cell line derived from A/J mice. We have previously described the M002 HSV-1 in detail [2, 4]. In brief, the virus is an attenuated human herpes virus mutant deleted for both copies of the *γ*_1_-34.5 gene, restricting virus replication to tumor cells. A genetically concatenated copy of murine IL-12 (p35 and p40 subunits, connected by an IRES) cDNA was inserted into each of the *γ*_1_34.5 loci under an Egr1 promoter. Cells infected with this virus make physiologic concentrations of IL-12, a potent antitumor cytokine that has been shown to enhance the cytolytic activity of natural killer cells and cytotoxic T lymphocytes as well as the development of a TH-1-type immune response [5–11]. IL-12 has also been shown to produce antiglioma immune activity in two different rodent models and also possesses antiangiogenic properties, an additional potential mechanism for antitumor activity [12, 13]. The combination of irradiated tumor cells, oncolytic virus, and local IL-12 production forms a multimodal vaccine approach to malignant CNS tumors that we now seek to apply to neuroblastoma.

In this study, we tested this vaccine against the syngeneic mouse tumor model, Neuro 2a neuroblastoma cell line, in A/J mice in both a postimplantation and a premunition prime/boost vaccination strategy. We also examined whether this vaccination strategy could produce a disease-specific sustained immune response to a rechallenge of the same tumor and whether* in vitro* cytotoxicity against tumor could be demonstrated from spleen cells of vaccinated mice.

## 2. Methods

### 2.1. Study Design and Vaccination Schedule

The study examined potential cancer vaccine settings that address combination therapy with oncolytic virus. Two groups of A/J mice (*n* = 10 per group) were vaccinated with either irradiated Neuro 2a whole cell (control vaccine (CV)) alone or the complete multimodal vaccine (CV-M002) manufactured as described below. Three control groups received no treatment (tumor control), M002 virus intracranial injection (virus control 1), or M002 virus intracranial injection followed by control vaccine injections (virus control 2) (Figure 2(a)). Mice received 1 × 10^6^ vaccine cells (in 50 *μ*L aliquots) at three, seven, and fourteen days following tumor implantation, inoculated into the gastrocnemius muscle. Vaccination was repeated as described for each experiment, using the contralateral gastrocnemius muscle from that previously used. Mice were followed up to determine an overall survival. A separate series of studies were undertaken involving vaccination with CV-M002 in mice with intracranial N2a tumors to evaluate tumor infiltration by immune-related cells.

In premunition experiment, a prime-boost strategy was employed (Figure 3(a)) whereby the first dose of complete multimodal vaccine or control vaccine was administered intramuscularly seven days prior to tumor placement and the second dose seven days after. All conditions were compared to an equivalent number of untreated tumor-bearing mice.

The rechallenge with syngeneic related (N2a) or unrelated (H6) tumor cells was performed in mice surviving initial N2a tumor implantation after receiving prime-boost vaccination with multimodal CV-M002. This group was challenged again with N2a cells at day 40 after the first N2a challenge and at day 60 with syngeneic H6 tumor cells to investigate specificity of immune response.

### 2.2. Mice, Tumor Cell Lines, and Virus

A/J mice, 6–8 weeks old, were purchased from Jackson Laboratories (Bar Harbor, Maine, USA). Mice were housed in a pathogen-free environment in the AALAC-accredited Animal Resource Center at the University of Alabama at Birmingham (UAB). The UAB Institutional Animal Care and Use Committee approved all protocols specific to this study (APN#080607062). The Neuro 2a mouse murine neuroblastoma cell line was originally derived from a spontaneous tumor of the spinal cord in an A/J mouse. We purchased the cell line from the American Type Culture Collection (CCL 131, passage 171). The H6 murine hepatoma of A/J origin was purchased from Jackson Laboratory, DCTD Tumor Repository (MRI Bank #J-750). The M002 virus has been previously described. Cells were maintained in Dulbecco's Modified Eagle's Medium mixed 50 : 50 with Ham's Nutrient Mixture F-12 (DMEM/F12) supplemented with 7% fetal bovine serum (FBS) and 2.6 mM L-glutamine (complete medium).

### 2.3. M002 Infection, Vaccine Preparation, and IL-12 Assay

The complete multimodal vaccine (CV-M002) was prepared as follows: Neuro 2a cells were cultured overnight in 24-well plates at 6 × 10^5^ cells/well followed by incubation (37°C, 2 hours) with the M002 virus at an MOI of 5. Cells were washed with PBS and resuspended in complete medium and incubated for an additional 30 min. The plate was then irradiated (10 Gy) followed by a medium exchange and additional incubation (37°C, 24 hours). Control whole cell vaccine (CV) consisted of an identical preparation in which the confluent flask underwent incubation procedures that were the same as M002-infected cells and received the 10 Gy irradiation dose. Supernatants were harvested, clarified by centrifugation, and frozen (−20°C) for IL-12 analysis.

### 2.4. Murine IL-12 ELISA

Production of murine IL-12 by the recombinant M002 virus was quantified using a Mouse IL-12 p70 Quantikine ELISA Kit (R&D System, Minneapolis, MN, USA). Twelve well plates were seeded with 2.5 × 10^5^ cells per well for 24 hours and then treated as described above (in preparation of cell vaccine). The supernatants were collected at 4, 12, and 24 hr and analyzed by ELISA according to the manufacturer's protocol.

### 2.5. Intracranial Tumor Injection

Following harvest from log-phase growth in culture, Neuro 2a cells (1 × 10^4^ cells) were injected into the right caudate nucleus in 5 *μ*L of serum-free DMEM/F12 containing 5% methylcellulose to retard “settling” during injection. This dose was optimized by Parker et al. [2] to define a median survival of 14 to 25 days. Anesthetized mice (ketamine/xylazine) were immobilized using a stereotactic frame. Recovered mice were assessed daily for weight and neurologic impairment and were euthanized on a timed schedule as described below, if they either incurred a 20% weight loss from baseline or appeared moribund. If mice were found dead, death was recorded to end on the previous day.

### 2.6. Flank Tumor Studies

H6 syngeneic hepatoma cells at a concentration of 5 × 10^6^ cells in 200 *μ*L serum-free media were implanted in the flank of long-term survivors of a second intracranial injection of Neuro 2a (at day 60 after initial N2a introduction). Tumor size was measured daily in three dimensions and volume was calculated. Mice were sacrificed if ulcerations were seen or if tumor volume exceeded 25 mm^3^.

### 2.7. Monoclonal Antibodies and Flow Cytometry

Semiquantitative flow cytometric analysis of brain homogenate from mice euthanized at multiple time points after initial tumor implantation was performed as described by Hellums et al. [3]. Beginning at seven days after initial tumor implantation, one mouse was euthanized at 36–48 h intervals. Homogenized cerebrum was enriched for lymphocytes by density-gradient centrifugation. The lymphocyte-rich layer was aspirated, washed, and resuspended in PBS-containing Fc blocker, followed by directly conjugated monoclonal antibodies from the following panel: CD4 (clone GK1.5, Pharmingen), CD8 (clone 53-6.7, Pharmingen), CD3 (clone 17A2), TCR-*γδ* (clone GL3), and NK cells (NCAM 60). All mAbs were matched in a separate tube by appropriate isotype controls. After incubation (60 min, 4°C), cells were washed three times and fixed in 1% fresh paraformaldehyde prior to acquisition on an LSR flow cytometer equipped with FACSDiva software (BD Biosciences, San Jose, CA).

### 2.8. Cytotoxicity Assay

Ten days after the second vaccination with the Neuro 2a vaccine described above, spleens were harvested and splenocytes were isolated by density-gradient centrifugation. Control splenocytes were obtained from naïve mice. Neuro 2a targets were harvested from culture, counted, and labeled with PKH-26 (Sigma-Aldrich, St. Louis, MO, USA) as described by Fischer and Mackensen [14]. Vaccinated splenocytes and naïve splenocytes were incubated with PKH26-labeled Neuro 2a cells at 37°C for 4 h in effector to target cell ratios of 12.5 : 1, 25 : 1, 50 : 1, and 100 : 1. The vital dye To-Pro-3-Iodide (Molecular Probes/Invitrogen, Carlsbad, CA, USA) was added immediately prior to acquisition on the flow cytometer.

### 2.9. Immunohistochemistry (IHC)

Paraffin embedded sections of mouse brain were postfixed in neutral buffered formalin followed by antigen retrieval with Rodent Decloaker (Biocare Medical, CA, USA). Sections were blocked with avidin and biotin blocks for 20 min (Avidin Biotin Blocking Kit, Biogenex Laboratories) followed by the Rodent Blocker (Bio Care Medical, Richmond, CA, USA) and Fc receptor blocker (Innovex Biosciences) for 30 minutes at RT.

Sections were incubated with rabbit CD3 (Abcam) antibodies overnight at 4°C following secondary goat anti-rabbit antibodies (Biocare Medical, Richmond, CA, USA) for 30 min at RT. Sections were next developed with streptavidin-labeled peroxidase and Turbo DAB (Innovex Biosciences, Richmond, CA, USA) for 2–5 minutes and counterstained with hematoxylin.

### 2.10. Data Analysis and Statistical Procedures

Statistical analysis was performed in the UAB Comprehensive Cancer Biostatistical and Bioinformatics Shared Services Facility. Descriptive statistics were used to express data from cytotoxicity assays. The primary endpoint in this study was survival. Survival times from tumor induction and from vaccine therapy were recorded in days for each mouse. Log-rank tests (Mantel-Cox and Gehan-Breslow-Wilcoxon) were performed on the survival data to determine the level of significance for any differences observed in treatment groups at the 0.05 level of significance. The median survival times across replications are represented as the median survival for the group. Minimum and maximum of the Kaplan-Meier estimates are provided.

## 3. Results

We initially hypothesized that the treatment with N2a tumor cells transduced with HSV1 (M002) encoding murine IL12 will demonstrate enhanced antitumor activity compared to M002 alone (or serve as more efficient vaccination vehicle than virus alone).

**Figure 1:**
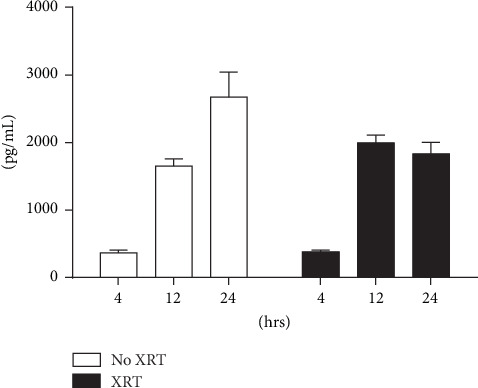
Murine IL-12 production in Neuro 2a cell line transduced with M002 virus. Murine IL-12 production was determined in nonirradiated and irradiated (10 Gy) Neuro 2a cell line transduced with M002 at MOI 5 pfu/cell. At 4, 12, and 24 hours after infection, concentrations of murine IL-12 in culture media were determined by ELISA. Data is reported as mean ± standard error of the mean. There was a significant increase in murine IL-12 production in untreated and XRT-treated cells as early as 12 hours after infection.

To characterize the N2a tumor cell-based vaccine loaded with the virus, we infected cells with M002 at MOI 5 pfu/cell and measured the production of murine IL12 in a culture media at 4, 12, and 24 hrs after infection. The mIL12 concentration in HSV-IL12 infected N2a cells increased in time and reached 3100 pg/mL at 24 hrs (Figure 1). An irradiation of M002-infected cells was implemented as* in vivo* required safeguard against the tumor cells proliferation. An irradiation at 10 Gy abolished cell amplification yet, however, still allowed cells to remain metabolically active for a certain time. In infected and irradiated (1 hr after infection) N2a cells, the production of mIL12 followed a similar pattern of the IL12 increase as detected in nonirradiated cells. Importantly, the IL12 levels were comparable with nonirradiated infected cultures.

Cellular vaccine presented by irradiated N2a cells loaded with HSV-IL12 was used in our study for peripheral (intramuscular) delivery of the mixture of tumor antigens, conditionally replicative HSV, and murine IL12 in an attempt to favorably change generally insufficient immune response to orthotopically implanted tumors.

**Figure 2:**
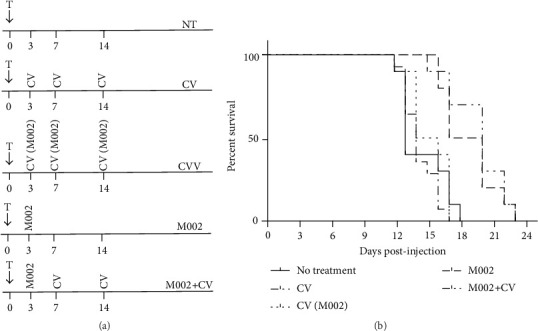
Survival of mice vaccinated after intracranial injection of Neuro 2a cells. (a) Treatment schema for five groups of A/J mice in survival experiment. A/J mice were stereotactically implanted in the right caudate nucleus with 1 × 10^4^ Neuro 2a cells. Ten mice per group were treated according to a schema at days 3, 7, and 14 after tumor cells implantation. Mice were vaccinated in the gastrocnemius muscle with 1 × 10^6^ irradiated Neuro 2a cells either sham infected (CV) or infected with M002 virus (CV-M002). In groups 4 and 5, M002 was injected intracranially at day 3 as single injection (group 4) or followed by vaccination prior to implantation. (b) Survival of mice in treatment groups.

Neuro 2a, one of the several clonal derivatives of the C-1300 spontaneous neuroblastoma of A/J mice, has been transplanted in various sites, including intracerebrally, for evaluating multiple therapeutic modalities. Macklis and Madison [15] implanted C-1300 in the brains of mice, and we have modified this approach for this study principally to use survival as our primary indicator. For the* in vivo* test of vaccine treatment, A/J mice were randomized into three cohorts: no treatment group, treatment with cell vaccine (CV), and treatment with CV (M002), complete multimodal cell vaccine. Vaccination was performed at 3, 7, and 14 days after tumor implantation (Figure 2(a), groups 1–3). In addition, two groups that received M002 viral treatment (groups 4-5) were included for comparison: both received single intracranial injection of virus at day 3, whereas the last group followed a boost with two intramuscular (IM) injections of CV. Contrary to our initial hypothesis,* in vivo* testing of HSV-IL12 loaded tumor cell vaccine did not result in any therapeutic benefit (increased survival) compared to either untreated group or vaccination with N2a tumor cells alone. In fact, neither the complete vaccine, a combination of irradiated N2a cell vaccine and M002 (CV-M002), nor the N2a cell vaccine alone (CV) conferred a significant survival advantage over untreated controls (Figure 2(b)). Mice that received three intramuscular injections of complete vaccine showed a median survival of 14 days and there was no advantage over 15 days for untreated tumor-bearing controls and 14.5 days for mice that received the N2a cell vaccine only (*p* = 0.7154 and 0.1261, resp.). There was no advantage for the complete M002 vaccine over the irradiated whole cell vaccine in this model (*p* = 0.7199). In these experimental settings, only viral treatment administered either as single intracranial injection at day 3 or followed by 2 N2a injections at days 7 and 14 modestly increased median survival time over untreated animals (20 days, 18.5 days, and 15 days correspondingly) (*p* = 0.0012). However, the combination of M002+radiated N2a cells provides no additional benefit over M002 alone (*p* = 0.6126).

Intracranial Neuro 2a tumors present an extremely stringent experimental model with low immunogenicity and rapid growth properties. Not surprisingly, both the whole cell vaccinated A/J mice and mice that received the complete M002 vaccine showed no survival advantage over that of tumor-bearing untreated mice as shown in Figure 2(b).

**Figure 3:**
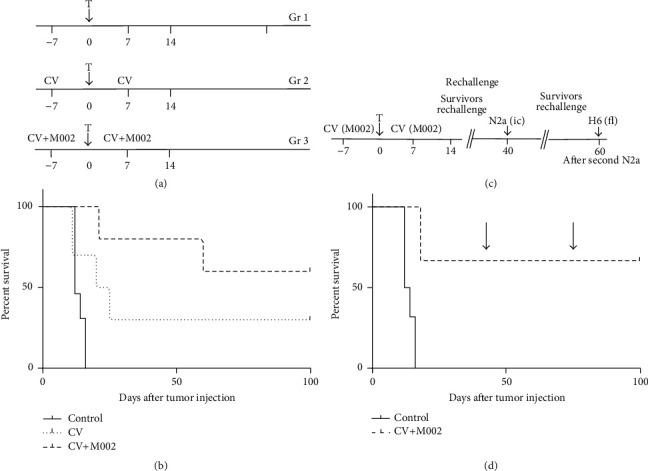
Survival of mice vaccinated before intracranial injection of Neuro 2a cells. (a) A/J mice (*n* = 10/group) were vaccinated with whole cell vaccine (CV) or M002-transduced cell vaccine (CV+M002) as in M&M seven days prior to intracranial implantation of 1 × 10^4^ Neuro 2a cells. The same vaccine boost was given seven days after tumor implantation. (b) Survival of mice in prevaccinated groups. All mice in control group died by day 16. Three mice survived in CV vaccinated group and six mice survived in CV+M002 vaccinated group at day 100 after tumor implantation. (c) A/J mice survivors of prevaccination experiment (*n* = 8) were rechallenged with 1 × 10^4^ Neuro 2a cells stereotactically implanted in the left (opposite) caudate at day 40. Control mice were injected in the same manner. (d) All control animals were dead by day 16. Vaccinated mice lived significantly longer than control mice (*p* = 0.0008). Five of six M002 vaccinated mice survived to the experiment endpoint.

In order to determine whether an effective adaptive antitumor response still could be induced with tumor cell vaccinations, a second experiment was conducted in which vaccination was performed at 7 days prior to tumor implantation followed by a boost at 7 days following tumor implantation (Figure 3(a)). A/J mice were randomized into three groups: the first consisting of tumor-bearing mice that were not vaccinated, a second cohort that received the whole cell Neuro 2a prime-boost vaccination regimen (CV), and a third that received the prime-boost vaccination with the complete CV-M002 vaccine. In contrast to vaccine treatment outcomes, a premunition prime-boost strategy conferred a survival advantage for vaccinated A/J mice despite rapid growth pattern of Neuro 2a tumors. A significant survival advantage was seen in mice that received the complete CV-M002 vaccine over that of control mice (*p* = 0.00002) and over that of mice that received the irradiated whole cell vaccine CV (*p* = 0.04). Survival of mice that received the whole cell vaccine was not significantly different from unvaccinated control mice (*p* = 0.12) as shown in Figure 3(b).

### 3.1. Surviving CV-M002 Vaccinated Mice Showed a Durable Immune Response

CV-M002 vaccinated survivors (*n* = 6) were rechallenged at day 40 with respective orthotropic tumor implantation in the contralateral hemisphere and compared with naïve mice that were implanted with the corresponding tumor as a positive control (Figure 3(c)). CV-M002-vaccinated mice lived significantly longer than control mice (*p* = 0.0008). Five of six M002 vaccinated mice survived to the experiment endpoint (100 days) (Figure 3(d)).

### 3.2. The CV-M002 Immune Response to the Vaccine Was Specific

Vaccine specificity was tested by flank injection of the syngeneic H6 hepatoma cells in CV-M002 surviving mice 60 days following the second intracranial Neuro 2a injection (Figure 3(c)). H6 tumor cells grew quickly in every mouse achieving a mass larger than 25 mm^3^ between seven and 10 days following the flank injections. Examination of brains from long-term survivors revealed no evidence of Neuro 2a tumor persisting from the second intracranial implantation. These data are consistent with specificity of* CV-M002 *vaccine against Neuro 2a tumors.

### 3.3. Analysis of Tumor-Infiltrating Lymphocyte (TIL) Phenotypes Reveals a Primary Adaptive T Cell Response

**Figure 4:**
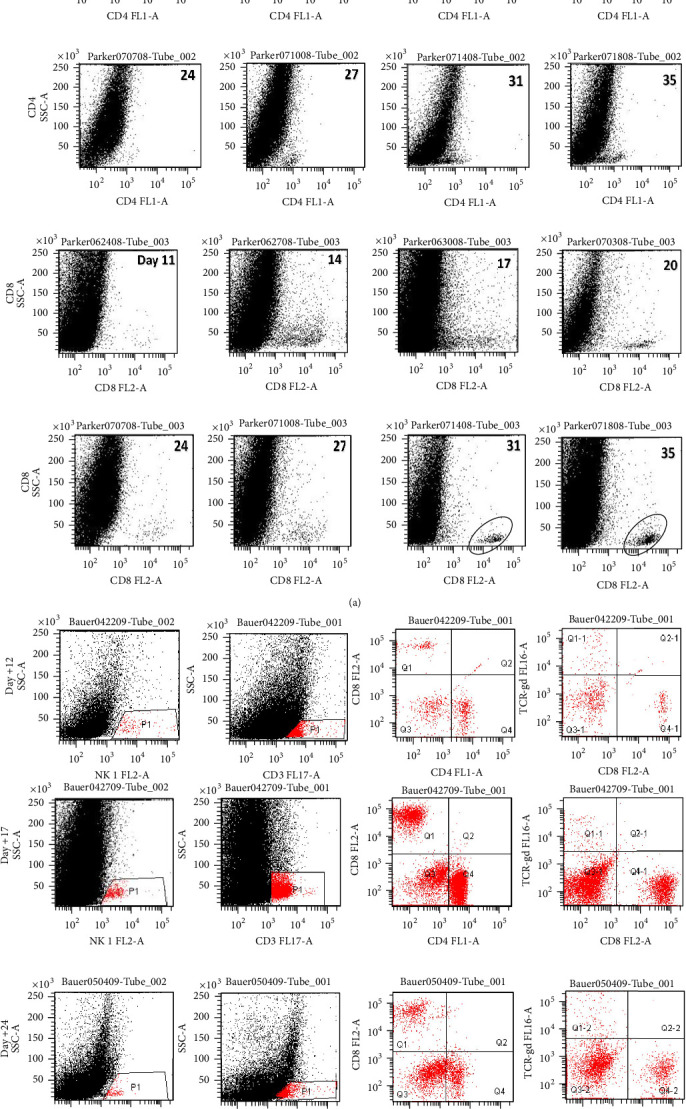
Increase in CD4 and CD8 LC in mouse brain in groups vaccinated before intracranial injection of Neuro 2a cells. A/J mice were vaccinated in the gastrocnemius with 1 × 10^6^ irradiated CV-M002 seven days prior to establishing brain tumors by stereotactically implanting 1 × 10^4^ Neuro 2a cells in the right caudate. Mice were serially euthanized, and the whole brain was prepared for flow cytometry analysis. (a) In this ungated acquisition of brain, the prevalence of CD4+ cells peaks between days 14 and 17 (top panel) and then decreases to a minimum by day 35 while a late response of CD8+ T cells is seen at day +31. (b) NK cell infiltration is small and with little change. T cells show significant infiltration at day +17 with the CD3+CD4+ subset tapering at day +24 as discussed above.

CV-M002 mice that received premunition vaccinations were serially sacrificed to evaluate tumor infiltration by immune cells. Beginning with day +7 after tumor injection and continuing every 3 days ×5, flow cytometric analysis of lymphocyte subsets was performed on brain/tumor-infiltrating lymphocytes at designated time points (Figure 4). One mouse from the nonvaccinated group (not shown) and one from the* CV-M002 *vaccine group were euthanized, and the brain underwent FACS analysis to evaluate TIL response using antibodies against CD3, CD4, CD8, and NK 1.1. An increase in CD4+ T cells was seen initially followed by a CD8+ T cell increase until reaching a peak at day +38 after tumor injection (Figure 4(a)). Analysis of NK cells and T cell subsets revealed that NK cell infiltration in the brain is small and with little change in time. All studied T cell subsets (CD3, CD4, and CD8) peaked in numbers at day 17 after tumor inoculation (Figure 4(b)). Thus, T cells show significant infiltration at day +17 with the CD4+ subset tapering at day +24.

### 3.4. Splenocytes from Vaccinated Mice Are Cytotoxic to Malignant Cells

**Figure 5:**
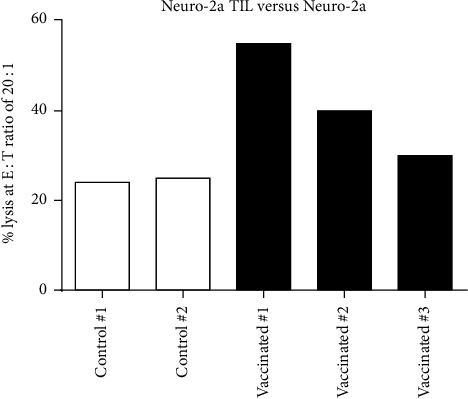
*In vitro* cytotoxicity of splenocytes of vaccinated and nonvaccinated mice against N2a. Neuro 2a cells are labeled with PKH-26 and incubated for 4 hours with splenocytes harvested from a vaccinated A/J mouse. To-Pro-3-Iodide is added just before flow cytometry analysis. Cell kill at E : T ratio of 20 : 1 is presented. Vaccinated mouse spleen cells are significantly more cytotoxic to Neuro 2a cells than controls 1 or 2 (*p* = 0.04).

Cytotoxic function of vaccinated and naïve mouse splenocytes was examined after a 4-hour incubation followed by the flow cytometry as described above. FACS analysis demonstrated a slight but significant (*p* = 0.04) increase in cytolytic activity of spleen cells from 2 of 3 vaccinated mice against Neuro 2a targets when compared with spleen cells from unvaccinated controls. At effector to tumor ratio 20 : 1, lysis of target tumor cells by vaccinated splenocytes reached 55%, 40%, and 30% compared to tumor cell lysis at 22% and 25% shown by naïve splenocytes (Figure 5).

### 3.5. CD3 Infiltration in Vaccinated Mice Brain Tumors

**Figure 6:**
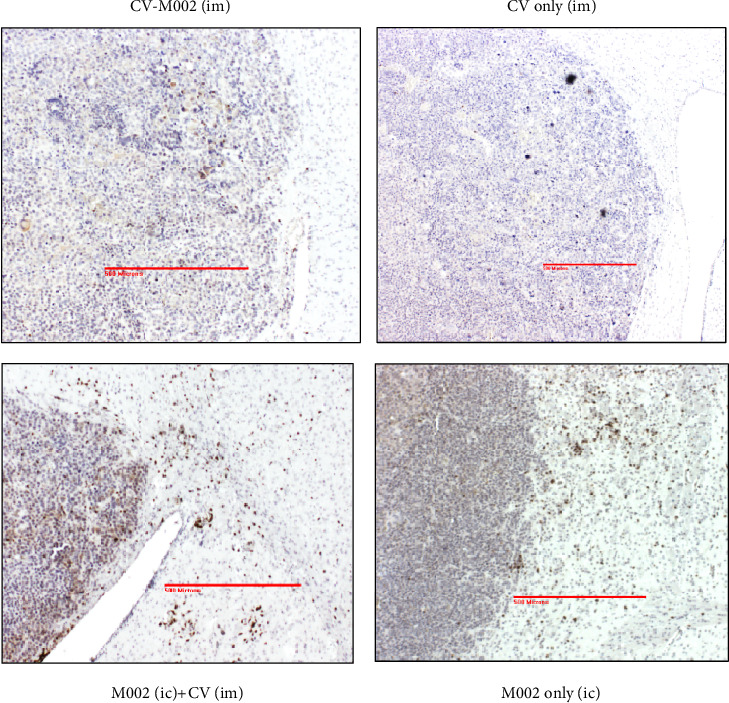
CD3 infiltration in mice brain tumors. Two groups of A/J mice (*n* = 10 per group) were vaccinated with either irradiated Neuro 2a control vaccine (CV) or the complete multimodal vaccine (CV-M002). Two control groups received intracranial injection of M002 virus followed by 2 injections of CV (M002 (IC)+CV (IM)) or single intracranial injection of M002 virus (M002 only (IC)). Beginning at day seven after initial tumor implantation, one mouse from each group was killed at 36–48 h intervals to harvest a brain for ICH. Paraffin embedded sections of brain tumors with adjacent normal brain were stained with anti-CD3 to detect lymphocyte infiltration. The most evident infiltrates were detected in both control and virus-injected groups. Some CD3 infiltration of tumors was seen in CV-M002, not CV only; however, it did not result in difference in overall survival for these two groups. All pictures were taken at 10x magnification. The scale bars in panels correspond to 500 microns.

Starting day 7 after initial tumor implantation, one mouse from each of the groups vaccinated with (a) complete multimodal vaccine (CV-M002) and (b) control vaccine (CV only) and of those receiving (c) intracranial injection of M002 virus followed by 2 injections of CV (M002 (IC)+CV (IM)) or (d) a single intracranial injection of M002 virus (M002 only (IC)) was killed at 36–48-hour intervalsto harvest a brain for ICH. Paraffin embedded sections of brain tumors with adjacent normal tissues were stained with CD3 AB to detect lymphocyte infiltration (Figure 6). The most prominent infiltrates were detected in both virus-injected groups. Some CD3 infiltration was seen in the brain tumors of CV-M002 vaccinated, but not in CV only group; however, it did not result in difference in overall survival for these two groups.

## 4. Discussion

Tumor vaccines have been part of clinical trials for many cancers including prostate, renal cell, gastrointestinal stromal, lung, and breast [16]. These trials are based on promising laboratory studies that often use autologous tumor that has been sonicated, lysed, irradiated, and/or transduced with a gene therapy vector. Vaccines may require antigen-presenting cells (APC) such as dendritic cells (DC) to initiate a cytotoxic T lymphocyte (CTL) response supplemented with adjuvant immune modulators such as IL-12 or GM-CSF. Vaccine protocols often include a boost injection of the vaccine or of immunomodulators to create a durable immune response to tumor-associated antigens. Vaccine trials directed against high-grade brain tumors such as* glioblastoma multiforme* [17] have incorporated the use of dendritic cells pulsed with tumor lysate [18, 19], antibodies to tumor antigen EGFRvIII [20], irradiated tumor injected with immunomodulators [21], and adoptive transfer of immunity of expanded tumor-infiltrating lymphocytes [22]. These trials are based on multiple laboratory models of tumor vaccines that use varied mechanisms in an attempt to induce a specific, durable antitumor immune response.

In this report, we have demonstrated the efficacy of an antitumor vaccine utilizing autologous (in our case, syngeneic) tumor cells infected with an oncolytic HSV-1. We demonstrated durability and specificity of this response by showing resistance to intracranial rechallenge with the same Neuro 2a tumor cell line but susceptibility to the H6 syngeneic murine hepatoma. These findings were consistent with a durable specific immune response against Neuro 2a tumor.* In vitro* cytotoxicity assays also confirmed that vaccinated splenocytes killed Neuro 2a tumor cells. Serial FACS analysis of whole brain preparations at multiple time points following vaccination also revealed a robust CTL response to the tumor.

We chose oncolytic HSV for our vaccine studies because of its demonstrated ability to generate an antitumor immune response. We specifically selected M002, an HSV expressing-IL-12, to amplify a more robust T cell response to the tumor. Oncolytic HSV has been used as a primary antiglioma agent in G207 and 1716 trials [23, 24]. Both viruses are conditionally replicating HSV that were directly injected into glioblastoma tumors. Phase I/II trials of G207 and 1716 have produced some long-term survivors.

The mechanism of immunity in our experiment is currently under investigation. A vaccine composed of a nonreplicating HSV infected tumor that is irradiated and then presented to dendritic cells has been shown to produce a strong T cell response including the stimulation of tumor reactive IFN-*γ* secreting T cells and tumor reactive cytotoxic T cells [25]. IL-12 boosts the Th1 immune response and stimulates the production of IFN-*γ* and TNF-*α*. This combination of irradiated tumor cells infected with a conditionally replication-competent oncolytic HSV may produce a more robust immune response in the context of the stress/danger model of immunity as originally described by Matzinger [26, 27] based on the expression of stress-related antigens. Indeed, malignant and infected cells, including gliomas, express stress-related antigens that are ligands for NKG2D, which functions in the primary immune response of NK and some *γδ* T cells and is a coreceptor on CD8+ CTL [5–8, 28]. In addition, M002 virus expression of IL-12 in the local tumor environment amplifies the immune response as described by Parker et al. [2].

Finally, it is generally obvious that prime-boost strategies are impractical as a single approach for most malignancies and especially neuroblastoma as it is impossible to accurately predict its development and characterize a population that would benefit from a preventive vaccine. However, a prime-boost approach could be an important component of a combination approach in patients with minimal residual or undetectable disease provided that the patient's immune function could be maintained at a healthy baseline or augmented by immune modulators [9, 10] such as effector cytokines [11], checkpoint inhibitors [12, 13], and/or adoptive cell therapy [29].

In summary, we have shown that a tumor vaccine incorporating the M002 IL-12-expressing virus appears to produce a durable, specific immunization against an aggressive intracranial tumor, although vaccination in a minimal disease state has little if any efficacy. These findings strongly suggest that multimodal vaccine therapy could have an adjuvant role in combination with additional immune modulation for the treatment of high-grade neural tumors.

## References

[1] Maris J. M., Hogarty M. D., Bagatell R., Cohn S. L. (2007). Neuroblastoma. *The Lancet*.

[2] Parker J. N., Gillespie G. Y., Love C. E., Randall S., Whitley R. J., Markert J. M. (2000). Engineered herpes simplex virus expressing IL-12 in the treatment of experimental murine brain tumors. *Proceedings of the National Academy of Sciences of the United States of America*.

[3] Hellums E. K., Markert J. M., Parker J. N. (2005). Increased efficacy of an interleukin-12-secreting herpes simplex virus in a syngeneic intracranial murine glioma model. *Neuro-Oncology*.

[4] Parker J. N., Pfister L.-A., Quenelle D. (2006). Genetically engineered herpes simplex viruses that express IL-12 or GM-CSF as vaccine candidates. *Vaccine*.

[5] Eisele G., Wischhusen J., Mittelbronn M. (2006). TGF-*β* and metalloproteinases differentially suppress NKG2D ligand surface expression on malignant glioma cells. *Brain*.

[6] Friese M. A., Platten M., Lutz S. Z. (2003). MICA/NKG2D-mediated immunogene therapy of experimental gliomas. *Cancer Research*.

[7] Groh V., Rhinehart R., Randolph-Habecker J., Topp M. S., Riddell S. R., Spies T. (2001). Costimulation of CD8*αβ* T cell by NKG2D via engagement by MIC induced on virus-infected cells. *Nature Immunology*.

[8] Groh V., Wu J., Yee C., Spies T. (2002). Tumour-derived soluble MIC ligands impair expression of NKG2D and T-cell activation. *Nature*.

[9] Vanneman M., Dranoff G. (2012). Combining immunotherapy and targeted therapies in cancer treatment. *Nature Reviews Cancer*.

[10] Puzanov I., Milhem M. M., Minor D. (2016). Talimogene laherparepvec in combination with ipilimumab in previously untreated, unresectable stage IIIB-IV melanoma. *Journal of Clinical Oncology*.

[11] Ahlers J. D., Belyakov I. M., Terabe M. (2002). A push-pull approach to maximize vaccine efficacy: abrogating suppression with an IL-13 inhibitor while augmenting help with granulocyte/macrophage colony-stimulating factor and CD40L. *Proceedings of the National Academy of Sciences of the United States of America*.

[12] Baghdadi M., Jinushi M. (2014). The impact of the TIM gene family on tumor immunity and immunosuppression. *Cellular and Molecular Immunology*.

[13] Ali O. A., Lewin S. A., Dranoff G., Mooney D. J. (2016). Vaccines combined with immune checkpoint antibodies promote cytotoxic T-cell activity and tumor eradication. *Cancer Immunology Research*.

[14] Fischer K., Mackensen A. (2003). The flow cytometric PKH-26 assay for the determination of T-cell mediated cytotoxic activity. *Methods*.

[15] Macklis J. D., Madison R. D. (1991). Neuroblastoma grafts are noninvasively removed within mouse neocortex by selective laser activation of intracellular photolytic chromophore. *The Journal of Neuroscience*.

[16] Schlom J., Arlen P. M., Gulley J. L. (2007). Cancer vaccines: moving beyond current paradigms. *Clinical Cancer Research*.

[17] Selznick L. A., Shamji M. F., Fecci P., Gromeier M., Friedman A. H., Sampson J. (2008). Molecular strategies for the treatment of malignant glioma—genes, viruses, and vaccines. *Neurosurgical Review*.

[18] Yamanaka R. (2009). Dendritic-cell- and peptide-based vaccination strategies for glioma. *Neurosurgical Review*.

[19] Ardon H., Van Gool S., Lopes I. S. (2010). Integration of autologous dendritic cell-based immunotherapy in the primary treatment for patients with newly diagnosed glioblastoma multiforme: A Pilot Study. *Journal of Neuro-Oncology*.

[20] Paraskevakou G., Allen C., Nakamura T. (2007). Epidermal growth factor receptor (EGFR)-retargeted measles virus strains effectively target EGFR- or EGFRvIII expressing gliomas. *Molecular Therapy*.

[21] Plautz G. E., Barnett G. H., Miller D. W. (1998). Systemic T cell adoptive immunotherapy of malignant gliomas. *Journal of Neurosurgery*.

[22] Quattrocchi K. B., Miller C. H., Cush S. (1999). Pilot study of local autologous tumor infiltrating lymphocytes for the treatment of recurrent malignant gliomas. *Journal of Neuro-Oncology*.

[23] Markert J. M., Medlock M. D., Rabkin S. D. (2000). Conditionally replicating herpes simplex virus mutant G207 for the treatment of malignant glioma: results of a phase I trial. *Gene Therapy*.

[24] Harrow S., Papanastassiou V., Harland J. (2004). HSV1716 injection into the brain adjacent to tumour following surgical resection of high-grade glioma: safety data and long-term survival. *Gene Therapy*.

[25] Benencia F., Courrèges M. C., Conejo-García J. R., Mohammed-Hadley A., Coukos G. (2006). Direct vaccination with tumor cells killed with ICP4-deficient HSVd120 elicits effective antitumor immunity. *Cancer Biology and Therapy*.

[26] Matzinger P. (1994). Tolerance, danger, and the extended family. *Annual Review of Immunology*.

[27] Matzinger P. (2002). The danger model: a renewed sense of self. *Science*.

[28] Bryant N. L., Gillespie G. Y., Lopez R. D. (2011). Preclinical evaluation of ex vivo expanded/activated *γδ* T cells for immunotherapy of glioblastoma multiforme. *Journal of Neuro-Oncology*.

[29] Kandalaft L. E., Powell D. J., Chiang C. L. (2013). Autologous lysate-pulsed dendritic cell vaccination followed by adoptive transfer of vaccine-primed ex vivo co-stimulated t cells in recurrent ovarian cancer. *OncoImmunology*.

